# Immunometabolism of Innate Immune Cells in Gastrointestinal Cancer

**DOI:** 10.3390/cancers17091467

**Published:** 2025-04-27

**Authors:** Izabela Siemińska, Marzena Lenart

**Affiliations:** 1 Institute of Veterinary Sciences, University Center of Veterinary Medicine JU-AU, University of Agriculture in Krakow, Mickiewicza 24/28, 30-059 Krakow, Poland; 2 Department of Clinical Immunology, Institute of Paediatrics, Faculty of Medicine, Jagiellonian University Medical College, Wielicka 265, 30-663 Krakow, Poland

**Keywords:** immunometabolism, gastrointestinal cancer, colorectal cancer, innate immune cells

## Abstract

Cancer cells are often described as voracious consumers of nutrients, with glucose frequently cited as a key energy source; however, their metabolic plasticity allows them to adapt and utilize various substrates, including lipids and amino acids, to sustain growth and survival. However, the metabolic demands of immune cells within the tumor microenvironment (TME) are less commonly discussed despite their critical role in shaping the immune response. In this review, we explored the intricate interplay between immunometabolism and innate immunity cells in gastrointestinal cancers. We focused on how metabolic pathways, including glycolysis, fatty acid oxidation, and amino acid metabolism, drive the immunosuppressive functions of myeloid-derived suppressor cells (MDSCs) and tumor-associated neutrophils (TANs), tumor-associated macrophages (TAMs) and innate lymphocyte subsets such as NK cells. These cells contribute to a hostile immune landscape, supporting tumor growth and evasion from immune surveillance in a phenomenon of tumor-derived immunosuppression. Additionally, we investigated the influence of dietary interventions on the metabolic reprogramming of these immune cells, highlighting how nutrition can modulate the TME. Finally, we discussed emerging therapeutic strategies that target metabolic vulnerabilities in MDSCs, TANs, NK cells, and monocytes, offering a novel avenue for enhancing antitumor immunity. By dissecting these mechanisms, we aim to provide insights into how metabolic pathways can be harnessed to improve cancer treatment outcomes. This review underscores the importance of understanding immunometabolism not only as a driver of immune suppression but also as a potential therapeutic target in gastrointestinal cancer.

## Simple Summary

This review explores the connection between immunometabolism and innate immune cells in gastrointestinal cancers. While cancer cells consume large amounts of nutrients, immune cells in the tumor microenvironment also have unique metabolic demands that influence immune responses. The review examines how metabolic pathways such as glycolysis, fatty acid oxidation, and amino acid metabolism contribute to the immunosuppressive functions of myeloid-derived suppressor cells (MDSCs), tumor-associated neutrophils (TANs), tumor-associated macrophages (TAMs), and innate lymphocytes such as NK cells. These cells create a tumor-friendly environment, helping cancer evade immune attack. The review also discusses how dietary interventions can reshape immune cell metabolism and highlights emerging therapies targeting these metabolic weaknesses to boost antitumor immunity. Understanding these processes could lead to new strategies for improving cancer treatment by manipulating immune cell metabolism.

## 1. Introduction

Tumor progression is promoted by communication between the tumor and infiltrating immune cells via the secretion of cytokines, growth factors, and enzymes that remodel the tumor microenvironment (TME). Within tumor-infiltrating leukocytes (TILs), a crucial component consists mainly of cells exhibiting immunosuppressive functions, such as tumor-associated macrophages (TAMs), tumor-associated neutrophils (TANs), myeloid-derived suppressor cells (MDSCs), but also innate lymphocyte subsets that physiologically takes part in antitumor response, such as NK cells [1,2].

Although an extensive interest in immunometabolism association with immunosuppression emerged quite recently, the first implications of the role of metabolism in tumor progression were observed 100 years ago and are known as the “Warburg effect” [3]. The author reported that cancer cells prefer glycolysis rather than oxidative phosphorylation for glucose metabolism and that glucose is consumed in large amounts. Now, it become apparent that excessive glucose consumption is also characteristic of immune cells, predominantly myeloid cells [4], suggesting that potential therapeutic metabolic interventions do not have to focus only on tumor cells but may manipulate immune response as well.

Immunometabolism plays a central role in dictating the activation and function of innate immune cells within the tumor microenvironment of gastrointestinal cancers. Macrophages, dendritic cells, and natural killer (NK) cells undergo dynamic metabolic reprogramming in response to hypoxia, tumor-derived metabolites, and nutrient fluctuations, which directly influence their antitumor immune responses. In gastrointestinal cancers, this immunometabolic reprogramming is orchestrated by key hub genes and pathways, including HIF-1α, mTORC1, AMPK, PPAR-γ, AhR, and STAT3. HIF-1α, activated under hypoxic conditions, promotes glycolysis and the expression of cytokines such as IL-10 and TGF-β [5,6]. TORC1 enhances glycolytic metabolism and inflammatory responses but can also contribute to immunosuppression when persistently activated, particularly through its interactions with lipid metabolism and autophagy [7,8]. Conversely, AMPK activation supports antitumor immunity by promoting oxidative metabolism and inhibiting the Warburg effect [9]. PPAR-γ regulates lipid uptake and storage, influencing immune responses through modulation of cytokine expression and metabolic state [10,11]. AhR, activated by environmental and endogenous ligands, contributes to immune suppression by enhancing glycolysis and regulating tryptophan metabolism via the kynurenine pathway [12]. Lastly, STAT3, frequently activated by cytokines such as IL-6, promotes glycolytic activity, inhibits oxidative metabolism, and modulates lipid regulatory genes such as *SREBP1* [13].

In this review, we summarize the latest knowledge on the role of immunometabolism in regulating innate immune cell functions in gastrointestinal cancers, explore potential therapeutic targets, and discuss future perspectives for further research.

## 2. Tumor-Associated Macrophages (TAMs)

TAMs are the most abundant innate immune cell infiltration tumor and TME, playing an important role in immune suppression and tumor growth progression. TAMs, being a specific type of macrophages, originate from monocytes, both recruited from the blood and tissue monocytic precursors [14]. Macrophages differentiate from resting, naive M0 macrophages either to M1- or M2-type, depending on the microenvironment, which is generally considered pro- and anti-inflammatory, respectively [15,16].

TAMs were shown to regulate glycolytic metabolic processes of tumor cells [17], including gastrointestinal cancers. In pancreatic ductal adenocarcinoma (PDAC), lysosomal glycoprotein CD68-positive TAMs infiltration was associated with glycolysis, measured as FDG maximal standardized uptake (FDG SUVmax) in a mechanism of glucose transporter 3 (GLUT3) expression stimulation in PDAC cells by IL-8 secreted by TAMs. IL-8 stimulates STAT3 phosphorylation and its translocation into the nucleus, resulting in upregulation of *GLUT3* gene transcription by its promoter activation [18]. Interestingly, GLUT3 was shown before as an important factor required for M2 macrophage polarization. GLUT3-induced M2 polarization relies on IL-4/STAT6 activation independently from glucose transport [19]. Consistent with this phenomenon, He Z. et al. observed that, in gastric cancer, GLUT-3-dependant glycolysis program activation in macrophages by IL-13 secreted by YAP1-overexpressing tumor cells promotes M2 polarization. As a result, M2 macrophages promoted 5-fluorouracil (5-FU) resistance of tumor cells in a mechanism of CCL8 activation of JAK1/STAT3 signaling pathway in tumor cells [20]. Cancer progression and metastasis were also shown to be promoted by M2-like TAMs capacity to consume intratumoral glucose, which fuels hexosamine biosynthetic pathway-dependent O-GlcNAcylation. Glucose metabolism resulted in O-GlcNAcylation of the lysosome-encapsulated protease Cathepsin B, elevating its level in TAMs and promoting its secretion into TME [21]. Elevated Cathepsin B expression was already associated with cancer progression, including CRC [22].

On the other hand, gastrointestinal cancers showed increased expression of lactate, which resulted from hypoxia and acidosis, which were shown to correlate with elevated risk of tumor metastasis and poor prognosis [23]. In CRC, high lactate levels inhibit TAMs glucose consumption [24]. CRC in vitro studies, in which SW480 and HT29 cell lines were cultured under hypoxic conditions, showed that the cell supernatants containing elevated lactate concentration stimulated M2-like polarization of THP-1 macrophages. Inhibition of lactate both reversed the effect on macrophage polarization and inhibited tumor growth [25]. M2 polarization of macrophages by lactate is mediated through the AKT/ERK signaling pathway [26], and this mechanism was also shown in esophageal cancer [27]. Lactate stimulates high-mobility group box 1 (HMGB1) expression and secretion by macrophages, promoting ERK, EMT, and Wnt signaling pathways activation in tumor cells [25]. Others showed that lactate-stimulated M2 macrophages secreted CCL8 and promoted CRC proliferation and metastasis through the CCL8/CCR5/mTORC1 axis [26].

In cancer progression, a dysregulation of fatty acid (FA) metabolism associated with FA oxidation (FAO) is frequently observed. The stimulation of peroxisome proliferator-activated receptor γ (PPAR-γ) was shown to increase FA metabolism in macrophages isolated from tumors in a murine model, resulting in their polarization towards M2-type [28]. Others showed, using a co-culture model of colorectal cancer (CRC) cells and macrophages, that upregulation of FAO in macrophages is associated with the expression of *ALKBH5* and *CPT1A* genes. ALKBH5 enhances fatty acid metabolism and M2 polarization of macrophages by upregulating CPT1A, promoting CRC development [29]. In this study, *CPT1A* gene expression stimulation is mediated by removing m6A (N6-methyladenosine) modification, which is a reversible RNA epigenetic modification that regulates RNA processing, shown to take part in tumor development and progression [30].

Miao H. et al. showed that CRC-associated macrophages exhibit ectopic expression of AB-hydrolase containing 5 (*ABHD5*), which promotes tumor growth [31]. ABHD5 is a co-activator of adipose triglyceride lipase (ATGL), which plays a crucial role in the lipolysis of triglycerides into diglycerides and free fatty acids [32]. ABHD5 suppresses spermidine synthase (SRM)-dependent spermidine production by inhibition of the reactive oxygen species-dependent expression of C/EBPɛ, which, in turn, activates *SRM* gene expression. Macrophage-specific *ABHD5* transgene-induced CRC growth in a subcutaneous xenograft mouse CRC model was suppressed when macrophages were transfected with additional *SRM* transgene [31].

What is more, tumor-derived lactate stimulates epigenetic modifications, in particular histone lysine lactylation (Kla), a phenomenon that was first documented by Zhang et al. in 2019, which occurs as a lactyl (La) group addition to the lysine amino acid residues of histone proteins [33]. Li X. M. et al. showed that, in CRC, tumor-derived lactate downregulates retinoic acid receptor γ (RARγ) expression in macrophages, which results in upregulation of IL-6 levels in the TME and macrophage tumor-promoting functions via TRAF6-IL-6-STAT3 signaling [24]. Moreover, very recent studies revealed an important role of histone lactylation and m6A methyltransferase METTL3 in KCNK6 in inflammation-associated carcinogenesis in mouse models of colon cancer. Increased Kcnk6 stability and upregulated expression of *Kcnk6* gene, in a YTHDF2-dependent manner, mediated by METTL3-mediated m6A modification and histone lactylation, respectively, increases NLRP3 inflammasome activation and inflammation-associated carcinogenesis [34].

In the case of amino acid metabolism, a crucial role seems to be played by tryptofan and its metabolism main sensor, the aryl hydrocarbon receptor (AhR). In PDAC, high AhR activity was associated with TAMs pro-tumor activity. *AhR* deletion or pharmacological inhibition the efficacy of immune-checkpoint blockage, infiltration of cytotoxic CD8^+^ T cells, and reduced PDAC growth, while TAMs immunosuppressive activity was downregulated by reduced tryptophan supplementation [35]. Moreover, in adenomatous polyposis coli (APC), a key tumor suppressor gene, deficient CRC, tryptophan 2,3-dioxygenase 2 (*TDO2*) was identified as an essential effector that, via Kyn-AhR pathway, upregulate glycolysis and anabolic cancer cell growth [36]. Single-cell RNA analysis of CRC patient samples revealed that the presence of highly expressing IDO1 macrophages, in comparison to low-IDO1 expressing macrophages, was associated with higher immune cell infiltration, higher immune checkpoint expression, and lower tumor progression [37].

**Figure 1:**
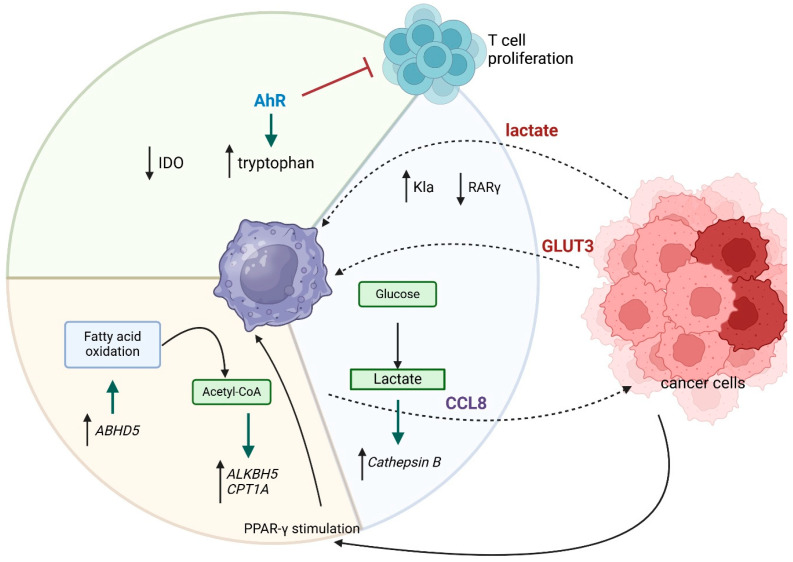
The impact of macrophage-tumor interactions on macrophage metabolism in gastrointestinal cancers. Tumor cells promote TAMs development via lactate secretion, GLUT3 production, AhR, and PPAR-γ stimulation, resulting in glucose, lipid, and amino acid metabolism, promoting further tumor growth and development. Arrows which are next to the protein names mean up- or downregulation.

In sum, in gastrointestinal cancers, tumor-derived factors, such as lactate, promote TAM polarization towards the pro-tumor M2 phenotype, which enhances tumor growth, metastasis, and resistance to treatment. We also discussed above how metabolic pathways such as glucose, lipid, and amino acid metabolism, including the key roles of GLUT3, PPAR-γ, and AhR, contribute to the tumor-promoting activities of TAMs. Moreover, recent findings on epigenetic modifications, such as histone lactylation and m6A methylation, add a new dimension to understanding how TAMs influence tumor progression through metabolic reprogramming. These insights underscore the complex metabolic network that fuels cancer development and the potential for targeting TAM metabolism as a therapeutic approach in gastrointestinal malignancies. The relationships discussed above are shown in Figure 1.

Apart from the common immunometabolic alterations observed across gastrointestinal cancers, the metabolic profiles of TAMs are also shaped by the distinct TME of CRC, PDAC, and gastric cancer. In both CRC and gastric cancer, TAMs primarily rely on glycolysis, with CRC, in particular, exhibiting a strong Warburg effect, leading to significant lactate accumulation that promotes an immunosuppressive phenotype and tumor progression [38]. In contrast, the hypovascularized stroma of PDAC imposes nutrient deprivation, compelling TAMs to favor oxidative phosphorylation (OXPHOS) over glycolysis to sustain their survival and pro-tumorigenic functions. Metabolic dependencies also diverge in terms of lipid and amino acid metabolism. In CRC and gastric cancer, lipid metabolism plays a pivotal role, with TAMs accumulating fatty acids that fuel their immunosuppressive state. However, in PDAC, amino acid metabolism dominates particularly glutamine metabolism. PDAC cells exhibit a high dependency on glutamine consumption, while TAMs actively contribute to this pathway by secreting glutamine-derived metabolites, further sustaining tumor growth and metabolic reprogramming [39]. Additionally, in CRC and gastric cancer, TAMs have been shown to upregulate IDO1, which converts tryptophan into kynurenine, a key immunosuppressive metabolite that dampens T-cell activation and promotes immune evasion. This mechanism is less prominent in PDAC, further emphasizing the metabolic divergence between TAMs in different gastrointestinal cancers. Overall, these observations suggest that TAM metabolism in CRC and gastric cancer shares notable similarities. Understanding these metabolic adaptations is crucial for designing metabolically targeted therapies that can selectively disrupt TAM-mediated immunosuppression in different GI cancers. 

## 3. Neutrophils

Neutrophils constitute the largest population of blood leukocytes and, such as various other immune cells, are highly heterogeneous. Circulating neutrophils can be categorized into normal-density neutrophils (NDNs) and low-density neutrophils (LDNs), the latter frequently identified in cancer as polymorphonuclear myeloid-derived suppressor cells (PMN-MDSCs). Tumor-associated neutrophils (TANs), analogous to macrophages, are further classified into TAN1 and TAN2 subsets based on their pro-inflammatory or immunosuppressive functions, respectively [40]. The neutrophil-to-lymphocyte ratio (NLR) has emerged as a prognostic marker in various cancers, including CRC, where elevated NLR correlates with poor survival [41,42].

Neutrophils metabolic activity undergoes significant changes during maturation. Progenitors and early precursors exhibit higher mitochondrial activity and primarily utilize fatty acid oxidation, the TCA cycle, and oxidative phosphorylation (OXPHOS) to meet their energy demands. In contrast, mature neutrophils demonstrate minimal dependence on mitochondrial respiration, favoring glycolysis as their predominant pathway for ATP generation [43]. Moreover, metabolism not only impacts neutrophil function but also neutrophils may act on the metabolism of cancer cells. Studies in CRC have shown that neutrophil extracellular traps (NETs) can induce tumor mitochondrial density, DNA, and ATP production and subsequently affect the growth of tumor cells [44]. In the hepatocellular cell carcinoma model, it has been shown that NETs formation by tumor-associated neutrophils is induced due to a metabolic switch into glycolysis and pentose phosphate pathway [45]. This mechanism was also confirmed in healthy circulating neutrophils [46]. Lactate, a glycolysis product, triggers the polarization of neutrophils into TAN2, which, via extracellular vesicles, enhances the proliferation of colorectal cancer cells [47]. Lactate metabolism by neutrophils has been linked to both colorectal cancer lesions and PDAC in mouse models and human studies, where its upregulation is associated with a shift toward an immunosuppressive phenotype, ultimately facilitating immune evasion [48,49,50].

Knowledge about the impact of amino acid metabolism on neutrophils is limited and only reveals that leucine may induce an antigen-presenting program in neutrophils, which is associated with favorable survival in most cancers [51]. In the case of the suppressive aspect role of arginase (ARG1), indoleamine-2,3-dioxygenase (IDO) and overall arginine metabolism by PMN-MDSCs will be as present lately. In PDAC, neutralizing ARG1 metabolism targets the NETs of TANs, which helps restore the activity of CD8+ T cells in the tumor microenvironment, ultimately enhancing the effectiveness of immune checkpoint therapies [52].

Tumor-associated neutrophils in patients with colorectal cancer have elevated gene levels and protein levels of molecules related to cholesterol metabolism, including the lipid transport receptor CD36 and lipoprotein receptor-related protein-1 (LRP1) and low-density lipoprotein receptor (LDLR) [53]. Moreover, cholesterol metabolism in the neutrophils was linked with the inhibition of NK cells by interrupting lipid raft formation and blocking antitumor signaling pathways [53]. Furthermore, lipid metabolism is also described as an essential way for TANs to survive under glucose-limited conditions [54].

In pancreatic cancer, a distinct population of TANs is characterized by metabolic reprogramming that enhances the TCA cycle and fatty acid metabolism. This shift boosts OXPHOS and amplifies the immunosuppressive activity of these cells also by upregulating PD-L1 expression [55]. Additionally, in PDAC neutrophil population characterized by relatively low glycolysis and a highly active citrate cycle was associated with an increased risk of liver metastasis [56].

**Figure 2:**
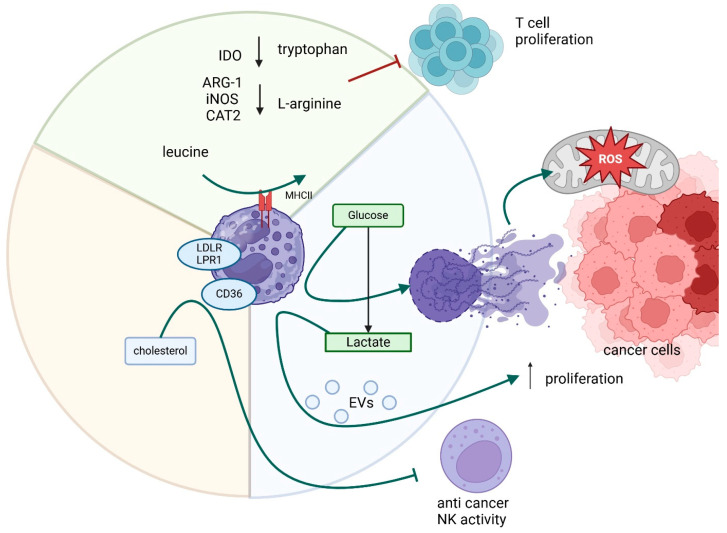
The impact of neutrophil metabolic shifts in gastrointestinal cancers. TANs suppress immune responses by metabolizing amino acids such as tryptophan and arginine, mainly by ARG1, IDO, and CAT2. They may be disrupting NK cell function through cholesterol metabolism through CD36, LRP1 and LDLR and promoting tumor growth via lactate-driven polarization into the immunosuppressive TAN2 phenotype by EVs. Moreover, fatty acid metabolism may be associated with increased PD-L1 expression and a higher risk of liver metastases. Additionally, glycolysis and NETs formation enhance tumor cell proliferation by increasing mitochondrial activity and ATP production. Arrows which are next to the protein names/processes mean up- or downregulation.

In CRC, neutrophil recruitment and NETs formation are inhibited by enoyl-CoA δ-isomerase 2 (*ECI2*), which is involved in lipid metabolism and reduces lipid-mediated interleukin 8 (IL-8) expression [57]. Neutrophil metabolism undergoes dynamic reprogramming in cancer, with both glycolysis and lipid metabolism contributing to immunosuppressive functions. Enhanced glycolysis supports NET formation and TAN2 polarization via lactate, promoting tumor growth and immune evasion in CRC and PDAC. Similarly, increased fatty acid oxidation and cholesterol metabolism sustain neutrophil activity in nutrient-deprived tumors and interfere with antitumor immune signaling. These metabolic shifts, characteristic of PMN-MDSCs and TAN2, represent key mechanisms by which neutrophils contribute to cancer progression and therapy resistance. The relationships discussed above are shown in Figure 2.

## 4. Myeloid-Derived Suppressor Cells (MDSCs)

Based on origin, the population of MDSCs is divided into three subpopulations, namely granulocytic/polymorphonuclear (PMN-MDSCs), monocytic (Mo-MDSCs), and “early-stage” MDSCs (e-MDSCs) [2]. Exhibit a range of immunosuppressive mechanisms, including the inhibition of T cell proliferation, induction of regulatory T cells (Tregs), and the polarization of various cell types, such as monocytes and neutrophils, into immunosuppressive phenotypes [58]. A significant increase in MDSCs was observed in several types of cancer, including gastrointestinal cancers, and this increase serves as a poor prognostic marker [59,60].

MDSCs exhibit an increased demand for and uptake of various metabolites, such as glucose, amino acids, and lipids, which are critical to sustaining their survival, immunosuppressive functions, and metabolic reprogramming within the tumor microenvironment. The immunosuppressive function of MDSCs associated with the development of CRC was linked with the intestinal fungus Candida tropicalis, which may enhance the uptake of glucose by MDSCs with simultaneous production of lactate and extracellular acidification rate (ECAR) level [61]. A similar observation was reported in gastric metaplasia induced by *Helicobacter*, where MDSCs exhibited high expression of genes such as Hexokinase (*Hk*), Triose Phosphate Isomerase (*Tpi*), Lactate Dehydrogenase (*Ldh*) and Glucose Transporter (*Glut*), all of which are involved in energy metabolism [62] which may also supply sufficient energy to support the immunosuppressive functions of MDSCs [63]. In PDAC, it has been shown that radiation may induce the activity of MDSCs due to lactate production [49], whereas inhibition of lactate production in a colorectal cancer mouse model led to a decreased presence of MDSCs but also other immunosuppressive cell populations such as regulatory T cells (Tregs) within the TEM [64].

MDSCs play a pivotal role in the metabolism of L-arginine, which is crucial for T-cell proliferation and represents a primary mechanism of their immunosuppressive activity. MDSCs enhance the expression of key enzymes, including ARG1 and inducible nitric oxide synthase (iNOS), while upregulating the cationic amino acid transporter 2 (CAT2) to effectively deplete L-arginine from the TME [65]. L-arginine depletion results in suppressed T-cell activity and a dampened antitumor immune response [66]. Similar influence on T cells have depletion of tryptophan where T-MDSCs in CRC express high IDO level responsible for tryptophan metabolism [67]. Amino acids such as arginine and glutamine are closely associated with MDSCs chemotaxis to tumor sites. Through the ERK/ETS2 signaling pathway, arginine can drive the transcription of CXCL1, a chemokine that acts as a chemoattractant for MDSCs by binding to the CXCR2 receptor [68].

In the case of lipid metabolism, it has been shown that tumor-infiltrating MDSCs (T-MDSCs) in colon cancer models increased fatty acid uptake and activated fatty acid oxidation. This was accompanied by an increased mitochondrial mass, upregulation of key FAO enzymes, and increased oxygen consumption rate [54]. Tumor-infiltrating MDSCs preferentially uses fatty acid β oxidation as a primary source of energy with displayed a significantly elevated expression of genes associated with FAO, including *CPT1*, *ACADM*, *PGC1β*, and *HADHA* [54]. Moreover, tumor-derived cytokines, such as G-CSF and GM-CSF, activate downstream signaling pathways, particularly STAT3 and STAT5, which drive the expression of lipid transport receptors such as CD36 on MDSCs. This upregulation enhances lipid uptake from the lipid-rich tumor microenvironment, thereby promoting the differentiation and accumulation of highly immunosuppressive human MDSCs in vitro [69]. These results underscore the pivotal role of lipid metabolism in shaping MDSCs function and their contribution to tumor immune evasion. In lipid metabolism is also involved another marker highly expressed on lectin-type oxidized LDL receptor 1 (LOX-1). Its expression on PMN-MDSCs of cancer patients was associated with response to endoplasmic reticulum (ER) stress, which seems to be a specific marker of human PMN-MDSCs [70], which may bind other ligands, including other modified lipoproteins, advanced glycation end products, aged red blood cells, apoptotic cells, and activated platelets [71]. In the context of lipid metabolism, it has been shown that decreased expression of miR-4435 promotes CRC tumorigenesis, in part by increasing the infiltration of PMN-MDSCs into the tumor microenvironment [72]. Moreover, fatty acid transport protein 2 (FATP2) facilitates the uptake of arachidonic acid and the synthesis of prostaglandin E2. Its selective inhibition not only suppresses the immunosuppressive activity of PMN-MDSCs and delays tumor progression but also enhances the efficacy of immune checkpoint inhibitors, positioning FATP2 as a promising therapeutic target for improving cancer treatment through the selective modulation of PMN-MDSCs function [73].

**Figure 3:**
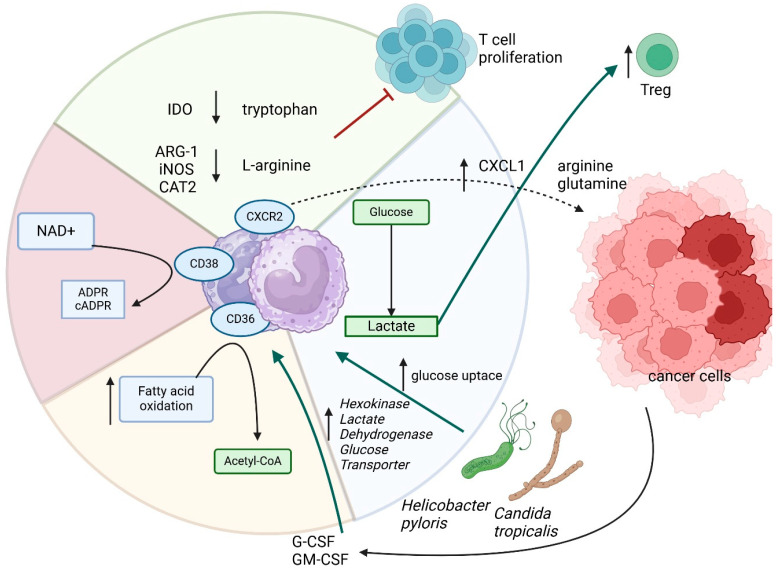
The metabolic adaptations MDSCs of in the tumor microenvironment of gastrointestinal cancers. MDSCs suppress immune responses by depleting key amino acids such as L-arginine and tryptophan, inhibiting T cell proliferation due to express ARG1, IDO, and CAT2. Pathogens such as *Helicobacter pylori* and *Candida tropicalis* further influence MDSCs metabolism, increasing glucose uptake and lactate production, which reinforces immunosuppression. Additionally, CD38-mediated NAD⁺ convention into ADPR and cADPR alters energy homeostasis, highlighting metabolism as a key driver of MDSCs function and tumor immune evasion. Arrows which are next to the protein names/processes mean up- or downregulation.

During the metabolism of lipids, carbohydrates, and amino acids vital coenzyme is NAD⁺. It participates in glycolysis and the Krebs cycle, acting as an electron carrier. In lipid metabolism, it is essential for beta-oxidation of fatty acids. In amino acid metabolism, it supports dehydrogenase reactions during their conversion to Krebs cycle intermediates. NAD⁺ can be regenerated through the mitochondrial electron transport chain or synthesized de novo from tryptophan or via salvage pathways using nicotinamide. This highlights its central role in energy production and metabolic regulation [74]. It seems that NAD^+^ is also important in the regulation of immune cells. In the case of MDSCs, CD38 catalyzes the conversion of NAD^+^ into ADP-ribose (ADPR) and cyclic ADPR (cADPR), which are pivotal for modulating cellular metabolic processes and signaling. The depletion of NAD^+^ by CD38 activity not only alters intracellular energy homeostasis but also reshapes the extracellular metabolite milieu within the tumor microenvironment [75]. An increased expression of CD38 was shown on Mo-MDSCs in advanced CRC penitents especially this one after therapy [76]. Whereas murine model esophageal cancer shows that MDSCs with CD38 expression poses more immature features [77]. Given its central role in MDSCs metabolism and immune suppression, CD38 represents a promising therapeutic target for reprogramming the tumor microenvironment and restoring antitumor immunity. Moreover, metformin-induced activation of 5′AMP-activated protein kinase (AMPK) decreased the MDSCs and M2 macrophage fractions by downregulating the mevalonate pathway [78]. Mevalonate production is an essential biosynthetic step that provides the precursors for de novo synthesis of cholesterol, which acts as a precursor for various membrane signal transduction and several other cellular components [79]. Taking it all together, glycolysis and lactate production drive their expansion, while lipid uptake and FAO sustain MDSCs survival and immunosuppressive activity. The relationships discussed above are shown in Figure 3.

## 5. NK Cells

NK cells play a key role in an anticancer immune response, being able to directly recognize and kill malignant cells. It is widely known that cancer cells and tumor microenvironment, including gastrointestinal cancers, impair NK cell activity as a part of the immune evasion process [80,81,82,83]. NK cell functional inhibition was associated with metabolic alterations, yet still little is known about NK cell immunometabolism in gastrointestinal cancers.

Glycolysis was proved to be essential for NK cell activation and, in hypoxic TME conditions, is driven by HIF-1α as overexpression of HIF-1α expression boosts glycolysis and NK cell activity in vitro and in vivo [84].

Tumor-derived lactate’s role in NK cell function seems indirect, relying mostly on the regulation of other cell subsets that can mediate NK cell dysfunction in TME, such as MDSCs or Treg cells [85,86]. The study of Ge W. showed, though, that NK cell’s functional impairment might be directly associated with lactate via sine oculis homeobox homolog 1 (SIX1)/lactate dehydrogenase A (LDHA) axis in pancreatic cancer. SIX1 expression was overexpressed in pancreatic cancer clinical samples, while NK cell co-culture with SIX-1 overexpressing pancreatic cancer cell lines resulted in downregulation of activation and cytotoxic markers in NK cells. NK cell impairment was reversed by the treatment of tumor cells with LDHA inhibitor and lactate transporter blocker [87].

NK cell defective lipid metabolism was shown to inhibit NK cell killing and dysregulate cytokine production in patients with inflammatory bowel disease, and this phenomenon was associated with aberrant mTORC1 activity. The assessment of mitochondria functions and glycoses revealed a distinct NK cell bioenergetic program relying mainly on the latter [88]. Sheppard S. et al. [89] showed that mice challenged with MHC class I-negative RMA-S lymphoma increased the uptake of fatty acids and the expression of carnitine palmitoyltransferase I (CPT1A). CPT1A is a key enzyme involved in fatty acid oxidation (FTO) in mitochondria, being involved in preventing long-chain fatty acid entry into the mitochondria [90]. CPT1A is required for NK cell cytotoxicity in a mechanism associated with cytoskeleton rearrangements, as CPT1A ablation reduces cytotoxicity due to impaired immune synapse formation [89].

FTO and CPT1A’s key role in NK cell metabolism was also shown in studies of the effect of fasting on NK cell anticancer activity. In the murine model of colorectal adenocarcinoma, cyclic fasting diet caused upregulation of glucocorticoids that mediate upregulation of CPT1A expression and fatty acids oxidation in spleen NK cells, although FA uptake by the cells was unaffected. Cpt1a^−/−^ NK cells were unable to control tumor growth during fasting when compared to WT NK cells, which observation was associated with low Cpt1a^−/−^ NK cells tumor infiltration. During fasting, NK cells were also redistributed from peripheral tissues to the bone marrow, and, in contrast to spleen NK cells, BM NK cells showed reduced FA uptake during fasting and downregulated CPT1A expression [91].

**Figure 4:**
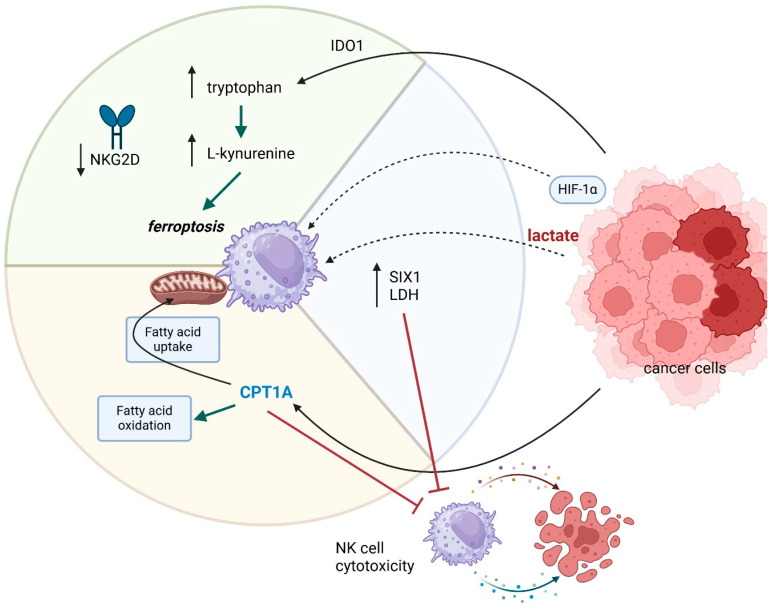
Metabolic alterations in NK cells mediated by gastrointestinal cancers. Tumor cells induce NK cell functional impairment via the production of HIF-1α, lactate, IDO, and CPT1A induction. Elevated tryptophan metabolism, AhR-independent, causes downregulation of crucial activating NK cell receptor NKG2D and ferroptosis, while CPT1A upregulates fatty acid uptake and metabolism. Arrows which are next to the protein names or processes mean up- or downregulation.

Amino acid metabolism of NK cells in gastrointestinal cancers is poorly understood. In gastric cancer, L-kynurenine, a metabolite of the amino acid l-tryptophan, mainly by IDO from tumor cells, impairs NK cells viability in TME, inducing ferroptosis in NK cells via an AhR-independent way [92]. Moreover, IDO1 induces NK cell dysfunction via downregulation of the activating receptor NKG2D on NK cells by inhibiting NKG2D ligand (NGD2DL) expression on lung cancer cells via the IDO1-Kyn-AhR signaling pathway [93]. Finally, IDO1 promotes tryptophan metabolism in the TME, recruiting and activating MDSCs, which, in turn, limits the availability of tryptophan for NK cells, causing their apoptosis [94]. The relationships discussed above are shown in Figure 4.

In sum, NK cells play a central role in antitumor immunity by directly recognizing and killing malignant cells; however, their activity is often suppressed in the TME of gastrointestinal cancers as part of immune evasion mechanisms. Recent studies have highlighted the importance of metabolic pathways such as glycolysis, fatty acid oxidation, and amino acid metabolism in regulating NK cell activation, cytotoxicity, and survival. However, much of this knowledge remains fragmentary. Glycolysis, lipid metabolism, and amino acid pathways all play important roles in regulating NK cell function within the tumor microenvironment. Factors such as hypoxia, tumor-derived metabolites, and immune-suppressive signals can disrupt these metabolic processes, leading to reduced NK cell activation and cytotoxicity. Altered fatty acid and tryptophan metabolism, in particular, have been linked to impaired NK cell responses in gastrointestinal cancers.

However, many aspects of NK cell immunometabolism in gastrointestinal cancers are unexplored. First of all, there is a notable lack of studies utilizing human tumor samples or patient-derived NK cells. Additionally, while research has predominantly focused on glycolysis and fatty acid oxidation, other metabolic pathways, such as amino acid metabolism, mitochondrial dynamics, and cholesterol metabolism, are underexplored in this context. For instance, the depletion of amino acids such as arginine and tryptophan in the tumor microenvironment has been shown to impair NK cell proliferation and function, highlighting the need for deeper investigation into these pathways [95]. Furthermore, mitochondrial fragmentation has been observed in tumor-infiltrating NK cells within human liver cancers, correlating with reduced cytotoxicity and poor patient prognosis [96]. Future studies should focus on (1) comprehensively profiling NK cell metabolism within gastrointestinal tumor tissue using advanced single-cell multi-omics approaches, (2) investigating how metabolic interventions, such as dietary modulation or metabolic inhibitors, may synergize with existing immunotherapies, and (3) exploring strategies to modulate mitochondrial dynamics to enhance NK cell function and tumor infiltration. Addressing these areas could pave the way for novel NK cell-based metabolic immunotherapies in gastrointestinal cancers.

## 6. Dendritic Cells

Dendritic cells (DCs) are a metabolically active and functionally diverse group of antigen-presenting cells, crucial for initiating and regulating antitumor immune responses. Among them, we distinguish conventional DCs type 1 (cDC1) and type 2 (cDC2), plasmacytoid DCs (pDCs), as well as monocyte-derived DCs (moDCs), each with distinct metabolic profiles and immunological roles. Among dendritic cells, we distinguish conventional DC type 1 (cDC1) and type 2 (cDC2), plasmacytoid DCs (pDCs), as well as monocyte-derived DCs (moDCs). Antitumor immunity of cDC1 has been emphasized by various studies, as well as colorectal cancer and pancreatic cancer [97,98]. DC2 represents a more heterogeneous population with a complex and still poorly defined role, especially in gastrointestinal cancers. In PDAC, cDC2 showed metabolic reprogramming, characterized by increased glycolysis and fatty acid metabolism, as well as increased activity in pathways related to hypoxia production of reactive oxygen species, which was associated with impaired antitumor immunity [99].

More recently, a higher frequency of tumor-infiltrating pDCs was associated with lower tumor stages and improved survival of colon cancer patients [100]. Moreover, higher level of blood-circulating pDCs was associated with longer survival of pancreatic cancer patients [101]. Mo-DCs, usually absent in a steady state, may arise from monocytes during inflammatory responses, including cancer, and drive antitumor immunity [102].

Glutamine plays a key role in enhancing CD8^+^ lymphocyte antitumor immunity through activation of DC1. However, in the colorectal cancer model, it was shown that glutamine availability is limited by competition between tumor cells and DC1, which use the SLC38A2 transporter for its uptake. This metabolic interaction affects the regulation of the immune response and may determine the efficacy of antitumor mechanisms [103]. Similarly, neutrophils, DC, and monocytes in colorectal cancer patient samples showed increased IDO expression [104]. Where increased expression of IDO in DCs can lead to the conversion of tryptophan to N-formylcine, which promotes the activation and function of Tregs, leading to suppression of the immune response [105].

**Figure 5:**
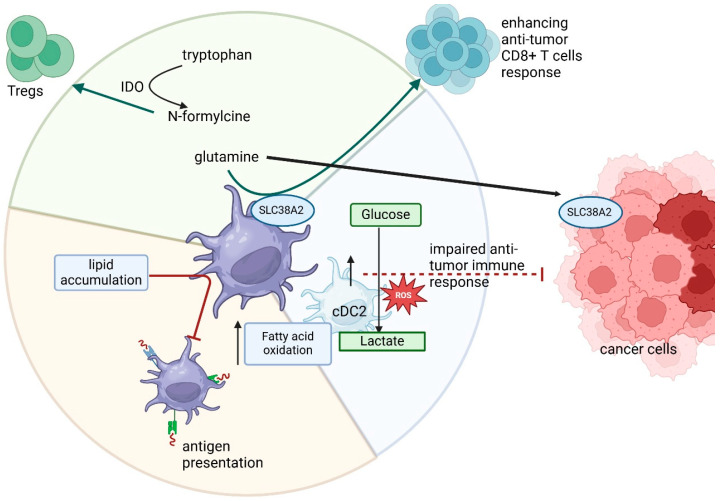
The metabolic changes in DCs in gastrointestinal cancers. The competition for glutamine between DCs and cancer cells may decrease the antitumor feature of cDC1, which enhances CD8^+^ T cells response. Moreover, excessive IDO expression cause tryptophan metabolism, resulting in an increase in Tregs activity. Additionally, lipid accumulation significantly affects the function of DCs and their ability to present tumor antigens. cDC2 shows metabolic reprogramming, characterized by increased glycolysis and fatty acid metabolism, as well as the production of reactive oxygen species, which is associated with impaired antitumor immunity. Arrows which are next to the protein names or processes mean up- or downregulation.

Additionally, oxidase lipid storage was observed in DC which impairs their ability to cross-present tumor antigens in mouse CRC cell lines [106]. Moreover, similar observations with restore of DC function and enhance the efficacy of cancer vaccines after pharmacological normalization of lipid level confirm another study [107]. All these findings suggest that the metabolic status of DC subsets has a critical impact on their ability to elicit effective antitumor responses. Abnormal metabolic reprogramming, whether through glutamine deficiency, tryptophan catabolism, or lipid accumulation, can impair antigen presentation and promote immunosuppression. Therefore, targeting DC metabolism may be a promising strategy to restore immune competence and improve the outcome of cancer immunotherapy. The relationships discussed above are shown in Figure 5.

## 7. Immunometabolism-Associated Therapy Approaches

Immunometabolism-associated therapy concerning innate immune cells is still unavailable, yet it might revolutionize future anticancer immunotherapy. At the moment, some potential therapeutics were suggested for macrophage, neutrophils, DCs and MDSCs metabolic alterations treatment (Table 1), while other populations, such as NK cells, require further studies.

Immunometabolism-associated might become a double-edged sword. For example, the usage of CPT1A as a potential target for cancer immunotherapy. CPT1A-targeted therapy was shown to boost radiation therapy sensitivity of nasopharyngeal carcinoma since CPT1A-Rab14 interaction promotes radiation resistance [111]. CPT1A overexpression was also observed in CRC cells, and its inhibition by the treatment with a secolignan-type compound, 2,6-dihydroxypeperomin B (DHP-B), a compound isolated from the plant Peperomia dindygulensis, suppresses tumor growth and progression [112]. However, CPT1A inhibition might have a negative impact on NK cell functions, as CPT1A upregulation promotes the anticancer activity of these cells. In the context of future therapies targeting the metabolism of immune cells in cancer, including gastrointestinal cancers, it is crucial to consider the safety and systemic implications of these interventions. The metabolic pathways targeted in immune cells, such as glycolysis and lipid and amino acid metabolism, are fundamental for the proper activity of not only immune cells but also numerous other cell types. Therefore, potential therapeutics may inadvertently affect non-immune tissues and organs, leading to side effects such as gastrointestinal mucosal injury, hepatotoxicity, and myelosuppression [113]. This is especially relevant in gastrointestinal cancers, in which the tumor microenvironment is characterized by metabolic competition and hypoxia. These conditions may impair T cell function and promote immune evasion [114,115]. Additionally, the overlap in metabolic dependencies between immune and tumor cells adds complexity—targeting pathways such as glutaminolysis or fatty acid oxidation may promote antitumor immune responses but also risk promoting tumor development [116]. For example, the monocarboxylate transporter 1 (MCT1) inhibitor lonidamine, investigated for its metabolic effects in cancer, has been associated with adverse effects, including alopecia and myelosuppression. This was demonstrated by Ning and Hahn, who reported the cytotoxicity of lonidamine both alone and in combination with other agents in murine and human cell lines [117]. Therefore, it is essential to develop selective immunometabolic therapies that minimize systemic toxicity while effectively enhancing antitumor immunity in gastrointestinal cancers. Moreover, inter-individual factors such as metabolic comorbidities and microbiome composition may further influence the safety and efficacy of these approaches [118], underscoring the need for precision strategies tailored to the unique metabolic landscape of the gastrointestinal tumor microenvironment. 

## 8. Conclusions

We are now starting to understand that immunometabolism plays a pivotal role in antitumor immune response and that targeting metabolic pathways opens new possibilities for cancer immunotherapy.

Promising approaches seem to be associated with dietary interventions, especially with fasting. It was shown that cyclic fasting might restore tumor-associated NK cell functional impairment, and, importantly, this observation comes from piloting the human study [91]. Fasting-mimicking diet impairs the pro-tumor functions of TAMs, especially under hypoxic conditions [119], as well as promotes protective gut microbiota, particularly *Lactobacillus johnsonii*, and increases CD45^+^ and CD8^+^ T cells, thereby suppressing tumor growth [120]. Clinical trials in colorectal cancer patients indicate that preoperative consumption of carbohydrate drinks reduced postoperative NLR values and the incidence and severity of postoperative complications following open colorectal surgery, compared with a preoperative fasting protocol [121]. In liver cancer, the blockage of glutamine uptake eliminates hydroxycarboxylic acid receptor 2, also known as GPR109A, and niacin receptor 1 (NIACR1) suppressive effect on myeloid cells [122].

Targeting MDSCs is particularly interesting, as numerous studies indicate that standard oncological treatments often remain ineffective against the MDSCs population [123]. In addition to the approach discussed in this study—modulating lipid metabolism to reduce the number or activity of MDSCs—inhibition of GLUTS, which is overexpressed on CD205⁺ PMN-MDSCs, has been shown to potentially restore antitumor immunity, as proved in a breast cancer model [124].

Interesting observations come from viral studies due to similarities in NK cell functional impairment in cancer and viral infections [125]. Retroviral studies proved that diet-related low iron levels lead to functional NK cell impairment, while iron supplementation enhances NK cell proliferation and their cytotoxic capacity [126,127]. NK cell nutrient uptake relies on the transcription factor IRF4, which is also important for the differentiation and expansion of cytomegalovirus-specific NK cells [128].

Finally, there are many understudied aspects of immunometabolism in gastrointestinal cancers that would be worth focusing on. For instance, in melanoma, the immunosuppressive activity of macrophages was associated with PERK signaling, which plays a crucial role in mitochondrial respiration and lipid oxidation [129], while NK cell functions and survival is inhibited by lactic acid [130]. Another highly understudied topic is an alteration of NK cell glucose metabolism in gastrointestinal cancers. Poznanski S. et al. showed that suppression of glycolysis in NK cells in ovarian cancer and their metabolic flexibility depending on TME in ovarian cancer [131].

In sum, further studies would help to address and overcome immunometabolic hurdles, supporting the development of effective gastrointestinal cancer immunotherapy.

## Figures and Tables

**Table 1:** Potential immunometabolism-associated targets and therapeutics for gastrointestinal cancer treatment.

Cells	Potential Target	Potential or Approved Therapeutic	Literature
Macrophages	ABHD5/SRM/spermidine axis	-	[31]
	IL-8/STAT3/GLUT3 signaling axis	reparixin	[18]
	RARγ-dependant TRAF6-IL-6-STAT3 signaling	nordihydroguaiaretic acid (NDGA)	[24]
	lactate transport function of MCT1	AZD3965, an MCT1 (monocarboxylate transporter 1) inhibitor	[108]
	AhR expression and M2-type polarization	JianpiJiedu decoction (traditional Chinese medicine)	[109]
Neutrophils	LRP1	LRP1 inhibitor	[53,110]
MDSCs	CD38	anti-CD38 monoclonal antibody (Daratumumab)	[77]
	activation of 5′AMP-activated protein kinase (AMPK) MDSCs	metformin	[78]
Dendritic cells	inhibitor of acetylCoA carboxylase	5(tetradecycloxy)2furoic acid (TOFA) in the research phase	[107]
